# Precision Medicine in Heart Failure: Integrating Ventricular–Vascular Interaction and Arterial Stiffness into Patient Phenotyping

**DOI:** 10.3390/jcm15093212

**Published:** 2026-04-23

**Authors:** Manuela Petrescu, Cristina Văcărescu, Cristina Tudoran, Stela Iurciuc, Dragoș Cozma

**Affiliations:** 1 Doctoral School, “Victor Babeș” University of Medicine and Pharmacy, 300041 Timisoara, Romania; narcisa.petrescu@umft.ro; 2 Research Center of the Institute of Cardiovascular Diseases, 13A Gheorghe Adam Street, 300310 Timisoara, Romania; cristina.vacarescu@umft.ro (C.V.); iurciuc.stela@umft.ro (S.I.); dragos.cozma@umft.ro (D.C.); 3 Cardiology Clinic, Institute of Cardiovascular Diseases, 300310 Timisoara, Romania; 4 Department of Cardiology, “Victor Babeș” University of Medicine and Pharmacy, 300041 Timisoara, Romania; 5 Department VII, Internal Medicine II, Discipline of Cardiology, “Victor Babeș” University of Medicine and Pharmacy, 300041 Timisoara, Romania; 6 Department of Cardiology, County Emergency Hospital “Pius Brînzeu”, 300723 Timisoara, Romania; 7 Centre of Molecular Research in Nephrology and Vascular Disease, “Victor Babeș” University of Medicine and Pharmacy, E. Murgu Square, Nr. 2, 300041 Timisoara, Romania; 8 Department of Internal Medicine and Cardiovascular Prevention, “Victor Babeș” University of Medicine and Pharmacy, 300041 Timisoara, Romania; 9 Department of Cardiology, Railway Clinical Hospital, 300643 Timisoara, Romania

**Keywords:** heart failure, arterial stiffness, pulse wave velocity, ventricular–vascular interaction, precision medicine, cardiovascular phenotyping, vascular aging

## Abstract

A key limitation in contemporary HF management is the marked heterogeneity of the syndrome, driven by diverse pathophysiological mechanisms that are not fully captured by traditional classifications based on left ventricular ejection fraction. Precision medicine has emerged as a promising approach to address this heterogeneity by integrating clinical characteristics, circulating biomarkers, advanced imaging, and computational phenotyping strategies. However, current frameworks predominantly emphasize myocardial dysfunction, while the contribution of vascular abnormalities remains underrepresented. The interaction between the left ventricle and the arterial system plays a fundamental role in cardiovascular performance. Arterial stiffness, commonly assessed by pulse wave velocity (PWV), represents a key determinant of vascular aging and a robust predictor of cardiovascular risk. Increasing evidence suggests that vascular dysfunction contributes significantly to the pathophysiology and clinical expression of HF, particularly in phenotypes characterized by preserved ejection fraction. This review synthesizes current evidence on precision medicine in HF and highlights the emerging role of arterial stiffness and PWV in multidimensional patient phenotyping. We propose that integrating vascular parameters into existing phenotyping frameworks may enhance risk stratification, improve mechanistic understanding, and support the development of more personalized therapeutic strategies in heart failure. Unlike previous reviews that have addressed arterial stiffness or heart failure phenotyping separately, this work uniquely integrates ventricular–vascular interaction and pulse wave velocity into a comprehensive precision medicine framework for heart failure. By bridging vascular physiology with data-driven phenotyping strategies, this review provides a novel conceptual model for incorporating arterial stiffness into multidimensional patient characterization across the full spectrum of heart failure phenotypes.

## 1. Introduction

Heart failure affects more than 64 million individuals worldwide and remains a leading cause of cardiovascular morbidity, mortality, and healthcare utilization [1,2,3,4]. Despite major therapeutic advances over the past decades, outcomes remain suboptimal, with high rates of hospitalization and mortality approaching 50% within five years of diagnosis [4]. The growing prevalence of heart failure reflects population aging, improved survival after acute cardiovascular events, and the increasing burden of cardiometabolic risk factors [2,4,5].

Historically, heart failure has been classified according to left ventricular ejection fraction (LVEF), leading to the widely used categories of heart failure with reduced (HFrEF), mildly reduced (HFmrEF), and preserved ejection fraction (HFpEF) [6,7]. Although this framework has important therapeutic and prognostic implications, LVEF alone provides only a partial representation of the biological complexity of the syndrome [8]. Patients within the same LVEF category often exhibit substantial variability in clinical characteristics, comorbidity profiles, hemodynamic patterns, and response to therapy [8,9].

Multiple biological pathways contribute to the development and progression of HF, including myocardial dysfunction, neurohormonal activation, systemic inflammation, metabolic disturbances, and vascular abnormalities [9,10]. This complexity partly explains the variability in therapeutic response observed in clinical practice and highlights the limitations of traditional classification systems. As a result, there has been growing interest in developing more sophisticated approaches for patient stratification and management.

Precision medicine has therefore emerged as an approach aimed at addressing this biological and clinical heterogeneity by tailoring diagnostic and therapeutic strategies to individual patient characteristics [8,11]. These approaches have been particularly informative in HFpEF, a condition characterized by marked heterogeneity and limited therapeutic options [8,12,13,14].

Despite these advances, vascular function remains insufficiently integrated into contemporary precision medicine frameworks in heart failure. Most current approaches prioritize myocardial structure, circulating biomarkers, and clinical variables, while the contribution of arterial properties is frequently overlooked [9,15]. Given the central role of ventricular–vascular interaction in determining cardiac performance, neglecting vascular function may result in incomplete characterization of heart failure phenotypes.

Arterial stiffness and pulse wave velocity represent key determinants of ventricular–vascular interaction and may contribute to the heterogeneity of heart failure phenotypes [16,17,18]. Pulse wave velocity (PWV) is widely regarded as the gold standard non-invasive method for assessing arterial stiffness [17]. Numerous studies have demonstrated that increased PWV is independently associated with cardiovascular events and all-cause mortality [17,19].

In the context of HF, growing evidence suggests that arterial stiffness and increased PWV contribute to disease pathophysiology, particularly in HFpEF, where vascular aging plays a central role [12,20,21].

**Figure 1:**
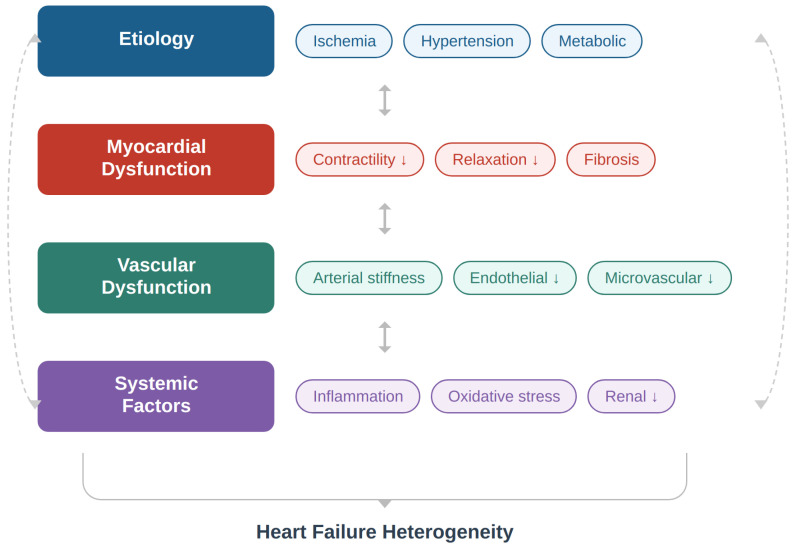
Determinants of heart failure heterogeneity. Heart failure heterogeneity arises from the complex interaction between etiological factors, myocardial dysfunction, vascular abnormalities, and systemic processes. These components interact bidirectionally and contribute to diverse clinical phenotypes through their combined effects on cardiovascular structure and function.

Unlike previous reviews that primarily focus on myocardial dysfunction, circulating biomarkers, or isolated aspects of heart failure phenotyping [6,8,12], the present review provides an integrated perspective that systematically incorporates vascular function—particularly arterial stiffness and pulse wave velocity—into precision medicine frameworks. This review uniquely emphasizes the role of ventricular–vascular interaction as a central pathophysiological mechanism linking myocardial performance with arterial load, thereby offering a more comprehensive understanding of heart failure heterogeneity. Furthermore, we highlight the translational potential of vascular markers in clinical practice, proposing their integration into multimodal phenotyping strategies that combine clinical, imaging, and computational approaches [20,22,23]. In this context, vascular function remains underrepresented in contemporary precision phenotyping strategies, and integrating pulse wave velocity into multimodal frameworks may improve risk stratification and support the development of individualized therapeutic strategies. A conceptual representation of these multidimensional mechanisms is presented in Figure 1.

Vascular functional markers—particularly measures of arterial stiffness such as pulse wave velocity—are rarely incorporated into integrated phenotyping models, despite their fundamental role in ventricular–vascular interaction and cardiovascular performance [2,3,4,24]. This review examines the role of vascular function in precision phenotyping of heart failure, with particular emphasis on arterial stiffness and pulse wave velocity. The following sections are organized to minimize conceptual overlap: Section 2 addresses the precision medicine framework, Section 3 focuses on the pathophysiology of ventricular–vascular interaction, Section 4 and Section 5 present clinical evidence and future directions, and Section 6 provides concluding perspectives. While mechanistic models of ventricular–vascular interaction have provided important physiological insights, their integration into precision phenotyping frameworks remains limited [2,6].

## 2. Precision Medicine in Heart Failure

### 2.1. Concept of Precision Medicine in Cardiovascular Disease

Precision medicine has emerged as a transformative paradigm in modern healthcare, aiming to tailor diagnostic and therapeutic strategies according to the biological and clinical characteristics of individual patients [11,22,25,26]. In contrast to traditional “one-size-fits-all” approaches, precision medicine integrates multiple sources of information—including clinical data, imaging findings, circulating biomarkers, genetic information, and environmental factors—to better characterize disease heterogeneity and guide individualized treatment strategies [22,25,27,28].

In cardiovascular medicine, the application of precision medicine has gained increasing attention, particularly in complex syndromes such as heart failure. HF represents a prototypical heterogeneous condition in which diverse etiological pathways converge toward a common clinical phenotype characterized by impaired cardiac function and reduced systemic perfusion [6,8,28]. This heterogeneity is reflected in differences in disease mechanisms, comorbidity profiles, hemodynamic characteristics, and therapeutic response among patients classified within the same clinical category [8,9].

Traditional HF management strategies have largely relied on guideline-directed medical therapy derived from large randomized clinical trials [6,7,29]. While these therapies have significantly improved outcomes in certain patient populations, particularly those with HFrEF, their benefits remain variable across the broader spectrum of HF phenotypes. In addition, many therapeutic interventions have demonstrated limited efficacy in conditions such as HFpEF, where underlying mechanisms are multifactorial and incompletely understood [6,12,21].

Precision medicine, therefore, seeks to refine patient stratification beyond conventional clinical parameters, enabling a more accurate identification of biologically meaningful subgroups of HF patients who may benefit from targeted therapeutic approaches [23,29,30].

### 2.2. Phenotyping of Heart Failure Patients

A central component of precision medicine in HF is the identification of disease phenotypes through advanced analytical methods. Phenotyping refers to the systematic classification of patients into subgroups based on shared biological, clinical, or pathophysiological characteristics [23,31,32].

Conventional phenotyping in HF has historically relied on clinical parameters such as LVEF, New York Heart Association (NYHA) functional class, and the presence of structural heart disease [6]. However, these variables provide only a limited representation of the complex biological processes involved in HF pathogenesis [8,33].

Recent advances in computational medicine have facilitated the development of data-driven phenotyping approaches that incorporate large multidimensional datasets. Techniques such as cluster analysis, machine learning algorithms, and unsupervised learning models allow researchers to identify patient subgroups based on patterns within clinical, biochemical, and imaging data [8,31,34,35].

One of the most influential studies in this field was conducted by Shah et al., who applied a phenomapping approach using unsupervised machine learning to identify distinct HFpEF phenotypes characterized by different clinical profiles, biomarker patterns, and outcomes [8]. These analyses revealed that HFpEF is not a single disease entity but rather a spectrum of biologically distinct phenotypes, each potentially requiring different therapeutic strategies.

Subsequent studies have confirmed that data-driven phenotyping approaches can improve risk stratification and may facilitate the identification of novel therapeutic targets [31,36]. Importantly, these approaches emphasize the need for integrative models that incorporate information from multiple physiological systems rather than focusing exclusively on myocardial function [17].

The identification of clinically meaningful heart failure phenotypes requires the integration of multiple sources of information, including clinical characteristics, circulating biomarkers, advanced cardiac imaging, and vascular function markers [23,30,35]. Recent developments in computational medicine and machine learning have further facilitated the identification of distinct patient clusters with different pathophysiological mechanisms and prognostic profiles [31,34,36]. A representative example of data-driven phenotyping in heart failure is provided by the phenomapping approach introduced by Shah et al. [8], which applied unsupervised machine learning techniques to identify distinct HFpEF subgroups. In this framework, multidimensional datasets—including clinical variables, imaging parameters, and circulating biomarkers—are integrated and analyzed using clustering algorithms to uncover latent patient phenotypes that are not apparent through conventional classification systems. Conceptually, this approach follows a stepwise process: first, comprehensive data acquisition across multiple domains; second, dimensionality reduction and pattern recognition using computational methods; and third, identification of phenotypic clusters with distinct pathophysiological profiles and clinical outcomes. Such models have demonstrated that HFpEF represents a heterogeneous syndrome composed of biologically distinct subgroups rather than a single disease entity [8,27]. Importantly, most existing phenomapping studies have focused predominantly on myocardial and systemic parameters, while vascular functional markers such as arterial stiffness and pulse wave velocity remain underrepresented. Integrating these variables into machine learning frameworks may further refine phenotypic classification and improve the identification of vascular-driven heart failure subtypes [8,31]. The principal domains contributing to precision medicine-based phenotyping in heart failure, along with their pathophysiological relevance, clinical utility, and methodological approaches, are summarized in Table 1.

Precision medicine approaches in heart failure integrate multiple biological and clinical domains, including patient characteristics, circulating biomarkers, cardiac imaging, computational phenotyping methods, and vascular functional markers, as summarized in Table 1 [23,30,35]. The combination of these complementary data sources enables improved characterization of disease heterogeneity and supports the identification of mechanistically distinct heart failure phenotypes [8,31,36,37].

### 2.3. Multimodal Approaches to Precision Medicine

The practical implementation of precision medicine in HF relies on the integration of several complementary diagnostic domains [23,30].

#### 2.3.1. Clinical Profiling

Clinical variables remain fundamental components of HF phenotyping. Factors such as age, sex, etiology of cardiomyopathy, comorbidities, and functional capacity contribute significantly to disease expression and prognosis [6,8]. For example, HFpEF frequently occurs in older individuals with multiple comorbidities, including hypertension, obesity, diabetes mellitus, and chronic kidney disease [12,13,14,38].

#### 2.3.2. Biomarkers

Circulating biomarkers provide valuable information regarding underlying biological processes in HF. Natriuretic peptides, including B-type natriuretic peptide (BNP) and N-terminal pro-B-type natriuretic peptide (NT-proBNP), remain the most widely used biomarkers for diagnosis and risk stratification [39]. In addition, emerging biomarkers reflecting myocardial injury, fibrosis, inflammation, and metabolic dysregulation are increasingly incorporated into multimodal risk prediction models [17].

#### 2.3.3. Cardiovascular Imaging

Advanced imaging techniques play a crucial role in precision medicine approaches. Echocardiography remains the cornerstone of HF evaluation, allowing detailed assessment of cardiac structure and function [6]. Novel echocardiographic techniques, such as myocardial strain analysis, provide insights into subclinical myocardial dysfunction [16].

Cardiac magnetic resonance imaging (CMR) offers additional information regarding myocardial tissue characterization, including the detection of fibrosis, inflammation, or infiltrative disease. These imaging modalities contribute to more refined phenotypic classification and may guide individualized treatment decisions [16,18].

Cardiac computed tomography (CT) has also emerged as a valuable complementary imaging modality in heart failure evaluation. Recent advances in functional and quantitative CT imaging—including late iodine enhancement and extracellular volume quantification—now allow myocardial tissue characterization comparable to CMR. Importantly, cardiac CT offers a viable alternative for patients who cannot undergo CMR due to the presence of non-MRI-conditional cardiac devices or claustrophobia, thereby broadening the applicability of advanced tissue characterization in clinical practice [34,40,41].

In addition to echocardiography and cardiac magnetic resonance, computed tomography has emerged as a valuable imaging modality for myocardial tissue characterization, enabling assessment of extracellular volume and late iodine enhancement [8,23,42]. This is particularly relevant in patients with contraindications to cardiac magnetic resonance imaging.

### 2.4. Limitations of Current Precision Approaches

Despite substantial progress in HF phenotyping, current precision medicine models remain incomplete. Most contemporary approaches emphasize myocardial structure and function, while relatively little attention has been given to the role of the arterial system [31,43,44,45].

However, cardiovascular performance depends not only on myocardial contractility but also on the mechanical properties of the arterial system. The interaction between the heart and the vascular system plays a critical role in determining ventricular loading conditions, cardiac efficiency, and overall circulatory performance [39].

Failure to account for vascular function may therefore limit the ability of existing phenotyping models to fully capture the pathophysiological complexity of HF.

### 2.5. The Emerging Role of the Vascular System

Increasing evidence suggests that vascular dysfunction represents a key component of HF pathophysiology [10,17,45]. Alterations in arterial properties may influence ventricular loading conditions, myocardial energy expenditure, and overall cardiovascular efficiency.

The concept of ventricular–vascular coupling describes the dynamic interaction between left ventricular contractility and arterial load [39]. Optimal cardiovascular performance requires a balanced relationship between these two components. Disruption of this balance may lead to impaired hemodynamic efficiency and contribute to the development and progression of HF.

Arterial stiffness, a hallmark of vascular aging, is increasingly recognized as an important determinant of cardiovascular outcomes [17,19]. Structural changes within the arterial wall—including elastin degradation, collagen accumulation, and vascular calcification—result in reduced arterial compliance and increased pulse pressure [17].

These vascular alterations may significantly influence cardiac workload and contribute to ventricular remodeling. Consequently, incorporating vascular parameters into HF phenotyping frameworks may enhance the ability to identify clinically meaningful subgroups of patients [17].

### 2.6. Toward Integrated Cardiovascular Phenotyping

Future precision medicine strategies in HF are likely to rely on integrated models that combine myocardial, vascular, and systemic parameters [23,30]. Such multimodal approaches may improve patient stratification, facilitate earlier detection of disease progression, and support the development of individualized therapeutic strategies [31,43].

In this context, the assessment of arterial stiffness and pulse wave velocity has emerged as a promising tool for evaluating vascular function and its contribution to HF pathophysiology. PWV provides a direct measure of arterial stiffness and reflects the mechanical properties of the arterial system [17,19].

Integrating vascular markers such as PWV into phenotyping algorithms may therefore represent an important step toward a more comprehensive understanding of HF heterogeneity and the implementation of truly personalized cardiovascular care [43,46].

**Figure 2:**
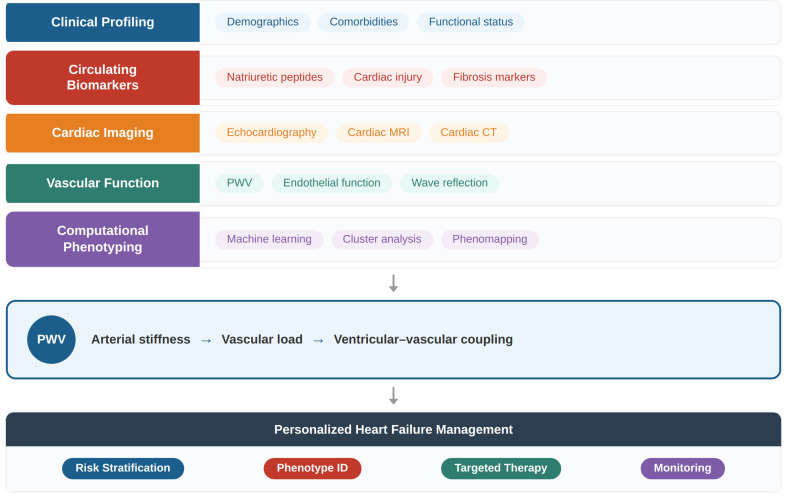
Integrated precision medicine framework in heart failure.

The transition from traditional heart failure classification toward precision medicine requires the integration of multiple biological domains, including myocardial, vascular, and systemic factors. In this context, arterial stiffness and pulse wave velocity may represent key components of an integrated phenotyping framework [44,47]. The conceptual model proposed in this review illustrates how vascular parameters may be incorporated into multidimensional patient characterization and personalized management strategies [Figure 2].

Multidimensional patient characterization integrates clinical features, circulating biomarkers, cardiac imaging, vascular function, and computational phenotyping approaches. Pulse wave velocity (PWV) serves as a key marker of arterial stiffness, linking vascular load to ventricular–vascular coupling and enabling improved risk stratification, phenotyping, and personalized management.

## 3. Ventricular–Vascular Interaction and Arterial Stiffness

### 3.1. Physiological Basis of Ventricular–Vascular Coupling

The cardiovascular system functions as an integrated hemodynamic unit in which the heart and the arterial system operate in close physiological interaction. The concept of ventricular–vascular coupling describes the dynamic relationship between left ventricular contractility and arterial load, two components that together determine overall cardiovascular performance [34,46,47].

From a hemodynamic perspective, ventricular–vascular coupling can be described by the relationship between ventricular elastance (Ees), which reflects myocardial contractile properties, and arterial elastance (Ea), which represents the effective arterial load imposed on the ventricle [34]. Optimal cardiovascular efficiency occurs when these two parameters are balanced, allowing the heart to generate adequate stroke volume while minimizing myocardial energy expenditure [47].

Under physiological conditions, the elastic properties of the large arteries play a crucial role in modulating the pulsatile nature of blood flow. During systole, the aorta and proximal elastic arteries expand in response to ventricular ejection, storing part of the stroke volume and mechanical energy. During diastole, the recoil of these arteries maintains forward blood flow and contributes to coronary perfusion. This buffering function of the arterial system, commonly referred to as the Windkessel effect, helps maintain stable hemodynamic conditions across the cardiac cycle [39].

However, alterations in arterial properties can disrupt this delicate balance between ventricular function and vascular load. Increased arterial stiffness leads to a rise in arterial elastance, thereby increasing the hemodynamic burden imposed on the left ventricle [17,47]. As a result, the ventricle must generate higher systolic pressures to maintain cardiac output, which may contribute to myocardial hypertrophy, increased oxygen demand, and progressive ventricular dysfunction.

**Figure 3:**
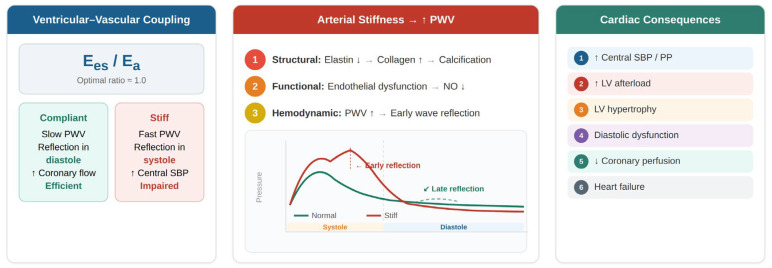
Ventricular–vascular coupling and the impact of arterial stiffness.

The interaction between left ventricular contractility and arterial load plays a fundamental role in determining cardiovascular performance. Structural alterations of the arterial wall increase arterial stiffness and accelerate the propagation of the pressure wave along the arterial tree. This leads to an earlier return of reflected waves during systole, resulting in increased central systolic pressure and augmented ventricular afterload [17,48]. These mechanisms contribute to impaired ventricular–vascular coupling and progressive myocardial dysfunction. The physiological relationship between ventricular function, arterial stiffness, and pulse wave velocity is illustrated in Figure 3.

Ventricular–vascular coupling reflects the interaction between ventricular contractility and arterial load, commonly expressed as the ratio between ventricular and arterial elastance (Ees/Ea). Increased arterial stiffness, reflected by elevated PWV, leads to earlier wave reflection and increased systolic load, impairing coupling efficiency and contributing to adverse cardiac outcomes.

### 3.2. Pathophysiology of Arterial Stiffness

Arterial stiffness reflects structural and functional alterations of the arterial wall that occur as a consequence of aging and exposure to cardiovascular risk factors [17,48,49,50]. These changes are primarily driven by progressive degeneration of elastin fibers, increased collagen deposition, vascular smooth muscle cell remodeling, and the development of medial calcification [43,48,49,51,52].

**Figure 4:**
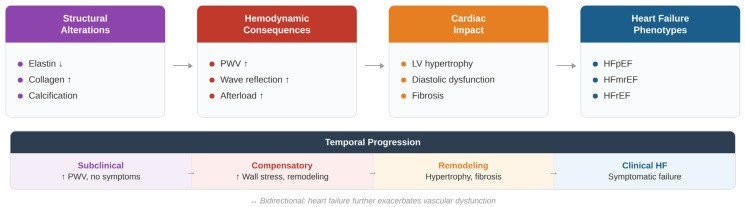
Pathophysiological mechanisms linking arterial stiffness to cardiac dysfunction.

Arterial stiffness has emerged as a key determinant of ventricular–vascular interaction and represents an important contributor to the pathophysiology and progression of heart failure [17,47,53]. Structural and functional alterations of the arterial wall—including elastin fragmentation, collagen accumulation, vascular calcification, and endothelial dysfunction—lead to reduced arterial compliance and increased pulse wave velocity [14,25]. The earlier return of reflected waves augments central systolic pressure and increases left ventricular afterload, thereby promoting myocardial hypertrophy, impaired ventricular relaxation, and progressive cardiac remodeling [47,48,54]. Over time, these hemodynamic and structural alterations contribute to the development and clinical progression of heart failure phenotypes [10,19]. The principal mechanisms linking arterial stiffness to ventricular dysfunction and heart failure development are summarized in Figure 4.

Structural and functional alterations of the arterial wall increase arterial stiffness and pulse wave velocity, leading to abnormal wave reflections and increased afterload. These hemodynamic changes promote left ventricular hypertrophy, diastolic dysfunction, and myocardial fibrosis, ultimately contributing to the development of heart failure phenotypes.

At the molecular level, several mechanisms contribute to vascular stiffening. Chronic inflammation and oxidative stress promote extracellular matrix remodeling within the arterial wall [51]. Activation of matrix metalloproteinases accelerates elastin degradation, while increased collagen synthesis leads to reduced vascular compliance [43,51]. In addition, endothelial dysfunction impairs nitric oxide bioavailability, further compromising the elastic properties of the arterial system [47].

These structural changes translate into important hemodynamic consequences. One of the most relevant effects of arterial stiffness is the accelerated propagation of the pressure wave generated during ventricular ejection [17,48,53]. In compliant arteries, the pressure wave travels relatively slowly, and reflected waves return to the aorta during diastole, contributing to coronary perfusion, whereas in stiff arteries, the pressure wave travels more rapidly, causing reflected waves to return earlier during systole [48,54].

The premature return of reflected waves increases central systolic blood pressure and pulse pressure, thereby augmenting left ventricular afterload. This phenomenon results in increased myocardial wall stress and contributes to ventricular hypertrophy, impaired diastolic relaxation, and progressive cardiac dysfunction [47,48].

### 3.3. Arterial Stiffness as a Determinant of Cardiac Remodeling

Growing evidence indicates that arterial stiffness plays a significant role in cardiac remodeling and heart failure progression. Increased pulsatile load imposed on the left ventricle contributes to structural changes in the myocardium, including hypertrophy, fibrosis, and alterations in ventricular geometry [19,48].

Experimental and clinical studies have demonstrated that increased arterial stiffness is associated with left ventricular hypertrophy and impaired diastolic function. These alterations may occur even in the absence of overt myocardial disease, highlighting the importance of vascular factors in cardiac remodeling [19,25].

The relationship between arterial stiffness and cardiac function is particularly relevant in the context of HFpEF. In this condition, patients frequently exhibit a phenotype characterized by advanced vascular aging, systemic hypertension, and increased arterial stiffness [50].

Moreover, increased pulsatile hemodynamic load may impair coronary microvascular perfusion, thereby contributing to myocardial ischemia and fibrosis. These processes further exacerbate ventricular stiffness and promote the development of heart failure symptoms [48,54].

### 3.4. Clinical Evidence Linking Arterial Stiffness and Heart Failure

Several clinical studies have demonstrated a strong association between arterial stiffness and adverse cardiovascular outcomes. Large observational studies have shown that increased arterial stiffness is an independent predictor of cardiovascular events, including myocardial infarction, stroke, and cardiovascular mortality [10,19].

In the context of heart failure, arterial stiffness has been associated with disease severity, exercise intolerance, and poor prognosis [40,55].

Importantly, the relationship between arterial stiffness and HF appears to be bidirectional. While vascular stiffening contributes to increased ventricular afterload and cardiac dysfunction, heart failure itself may further exacerbate vascular abnormalities through neurohormonal activation, inflammation, and endothelial dysfunction [47,48].

This complex interaction between myocardial and vascular factors highlights the need for integrated approaches to HF phenotyping that account for both cardiac and vascular components of the disease [12,25].

### 3.5. Implications for Precision Medicine

The recognition of arterial stiffness as an important determinant of cardiovascular function has significant implications for precision medicine in HF [23,30]. Incorporating vascular parameters into HF phenotyping models may help identify patient subgroups characterized by specific hemodynamic profiles.

For example, patients with HFpEF and pronounced vascular stiffness may represent a distinct phenotype driven primarily by abnormalities in ventricular–vascular coupling [9,25].

Furthermore, the assessment of arterial stiffness may provide additional prognostic information beyond traditional risk markers [19,55]. Integrating vascular parameters into multimodal diagnostic frameworks that include imaging, biomarkers, and clinical variables could improve risk stratification and support individualized patient management [12,14].

In this context, noninvasive measures of arterial stiffness—particularly pulse wave velocity—have emerged as promising tools for evaluating vascular function and its contribution to HF pathophysiology [17,19].

### 3.6. Limitations and Controversies

Despite the growing body of evidence supporting the role of arterial stiffness and pulse wave velocity in heart failure, several important limitations and unresolved controversies should be acknowledged [47,56,57,58,59,60].

First, substantial methodological heterogeneity exists across studies evaluating arterial stiffness, including differences in measurement techniques (e.g., carotid–femoral versus brachial–ankle pulse wave velocity), device calibration, and path length estimation. These variations limit comparability between studies and may affect the reproducibility of findings [17,48,55,61].

Second, the causal relationship between arterial stiffness and heart failure remains incompletely understood. While increased arterial stiffness contributes to elevated afterload and ventricular remodeling, heart failure itself may further exacerbate vascular dysfunction through neurohormonal activation and systemic inflammation, making it difficult to disentangle cause and consequence [9,43,57].

Third, the prognostic value of pulse wave velocity may vary across heart failure phenotypes. Although several studies suggest a stronger association in HFpEF, data in HFrEF remain less consistent, and the incremental value of PWV beyond established biomarkers and imaging parameters is still under investigation [47,56,57,59,60].

Finally, most available evidence derives from observational studies, and there is a lack of large-scale prospective and interventional trials evaluating whether targeting vascular stiffness can improve clinical outcomes [59,62]. Addressing these limitations will be essential for the integration of vascular parameters into precision medicine frameworks and for establishing their role in routine clinical practice.

## 4. Pulse Wave Velocity in Clinical Practice

### 4.1. Methods for Measuring Pulse Wave Velocity

Several techniques have been developed for the non-invasive assessment of PWV, with carotid–femoral pulse wave velocity (cfPWV) currently considered the gold standard for evaluating central arterial stiffness [17,61,63].

The cfPWV measurement reflects the stiffness of the thoracic and abdominal aorta, which are the major elastic arteries responsible for buffering pulsatile blood flow [25,43]. The measurement is typically performed by recording pulse waveforms simultaneously or sequentially at the carotid and femoral arteries using applanation tonometry, mechanotransducers, or oscillometric devices [51]. The time delay between the arrival of the pulse wave at these two sites is calculated and divided by the estimated arterial path length.

Consensus recommendations emphasize the importance of standardized measurement protocols in order to improve reproducibility and comparability between studies [51,61]. Factors such as patient positioning, blood pressure stabilization, and accurate measurement of the arterial path length are essential for reliable PWV assessment.

In addition to cfPWV, other PWV measurement techniques have been developed. Brachial–ankle PWV (baPWV) is widely used in large epidemiological studies, particularly in Asian populations [43]. Although baPWV is easier to measure, it reflects both central and peripheral arterial stiffness and therefore provides less specific information regarding aortic elasticity.

Recent technological developments have also led to the emergence of oscillometric devices capable of estimating PWV using single cuff measurements [49,54,61]. These devices are increasingly used in clinical settings due to their simplicity and operator independence, although their accuracy compared with tonometric methods remains an area of ongoing investigation.

### 4.2. Prognostic Significance of Pulse Wave Velocity

Over the past two decades, numerous prospective studies have demonstrated that increased PWV is strongly associated with adverse cardiovascular outcomes. PWV has been shown to predict major cardiovascular events, including myocardial infarction, stroke, and cardiovascular mortality, independently of traditional risk factors [19,59,64,65].

Meta-analyses have demonstrated that each 1 m/s increase in PWV is associated with a significant increase in the risk of cardiovascular events and all-cause mortality [54,66]. This relationship highlights the importance of arterial stiffness as a marker of vascular aging and cardiovascular risk.

The prognostic value of PWV extends beyond traditional cardiovascular disease populations. Elevated PWV has been observed in patients with hypertension, chronic kidney disease, diabetes mellitus, and metabolic syndrome, all of which are conditions associated with accelerated vascular aging [17,19,67].

In addition, PWV has been increasingly recognized as an important indicator of subclinical organ damage. Increased arterial stiffness contributes to microvascular injury in several organs, including the heart, brain, and kidneys [54,66]. As a result, PWV measurement may provide valuable insights into the systemic consequences of vascular dysfunction.

### 4.3. Pulse Wave Velocity in Heart Failure

The role of arterial stiffness in heart failure has attracted increasing attention in recent years. Elevated PWV values have been reported in patients across the HF spectrum, including both HFrEF and HFpEF populations [40,41].

In HFrEF, increased arterial stiffness may contribute to impaired ventricular ejection by increasing afterload and altering ventricular–vascular coupling [26]. Elevated arterial elastance increases myocardial workload and may exacerbate ventricular dysfunction, particularly in patients with already compromised systolic function.

In HFpEF, the contribution of arterial stiffness appears to be even more prominent. Patients with HFpEF often exhibit a phenotype characterized by advanced vascular aging, systemic hypertension, and increased arterial stiffness [9,18,25]. These vascular abnormalities contribute to increased pulsatile load, impaired ventricular relaxation, and elevated left ventricular filling pressures.

Furthermore, the early return of reflected waves in stiff arteries increases central systolic pressure and reduces diastolic pressure, potentially compromising coronary perfusion [48,54]. These hemodynamic alterations may promote myocardial ischemia, interstitial fibrosis, and progressive ventricular stiffening.

Clinical studies have shown that increased PWV in HF patients is associated with worse functional status, reduced exercise capacity, and increased risk of hospitalization and mortality [3,40]. These findings suggest that PWV may serve as a valuable marker of disease severity and prognosis in HF.

Several observational and cohort studies have investigated the prognostic role of pulse wave velocity in patients with heart failure. These studies consistently demonstrate that increased arterial stiffness is associated with adverse cardiovascular outcomes, impaired ventricular–vascular coupling, and increased mortality risk [40,41,64,68]. A structured overview of representative studies evaluating arterial stiffness and pulse wave velocity in heart failure, including study design and methodological approach, is presented in Table 2.

### 4.4. Integrating PWV into Precision Medicine

Despite the growing evidence supporting the role of PWV in cardiovascular risk assessment, its integration into precision medicine frameworks for HF remains limited [23,30].

Most current HF phenotyping strategies focus on myocardial structure, biomarkers, and clinical characteristics, while vascular parameters are less frequently included in risk stratification models [18,25]. However, given the central role of ventricular–vascular interaction in cardiovascular physiology, neglecting vascular function may result in incomplete characterization of the disease [17].

Incorporating PWV into HF phenotyping models could provide several advantages. First, PWV offers a direct and quantitative measure of arterial load, which may help identify patients with a predominantly vascular phenotype of HF. Second, PWV measurement may improve risk stratification by identifying patients at higher risk of adverse outcomes. Third, PWV could potentially serve as a marker for monitoring therapeutic interventions aimed at improving vascular function [3].

Future research should explore the integration of PWV with multimodal datasets that include imaging parameters, circulating biomarkers, and computational phenotyping approaches [23,31,34,36]. Such integrative models may allow the identification of novel HF phenotypes and support the development of more personalized therapeutic strategies.

## 5. Future Directions and Clinical Implications

### 5.1. Beyond Myocardial Dysfunction: A Systemic Perspective

Heart failure should increasingly be conceptualized as a systemic cardiovascular syndrome rather than an isolated myocardial disorder. While traditional paradigms have focused predominantly on ventricular dysfunction, accumulating evidence supports the critical contribution of vascular and systemic factors in shaping disease expression and progression [9,10,17,48].

Within this framework, arterial stiffness emerges as a central integrative marker reflecting the cumulative effects of aging, cardiometabolic burden, and vascular remodeling [17,49]. Incorporating vascular parameters into precision phenotyping models is therefore likely to improve the identification of mechanistically distinct patient subgroups and facilitate the development of targeted therapeutic strategies [23,30].

Within this integrative framework, the arterial system emerges as a critical determinant of cardiovascular performance. The mechanical properties of the arterial tree influence ventricular loading conditions, myocardial energy expenditure, and overall circulatory efficiency [17,43]. Consequently, vascular dysfunction may represent not only a consequence of cardiovascular disease but also an active driver of HF progression [47,48].

This paradigm shift has important implications for precision medicine strategies in HF. Traditional phenotyping approaches have focused predominantly on myocardial parameters such as LVEF, ventricular geometry, and biomarkers of myocardial injury or stress. However, these variables alone cannot fully capture the systemic nature of HF pathophysiology [9].

Integrating vascular parameters into HF phenotyping models may therefore provide a more comprehensive representation of disease mechanisms and allow identification of previously unrecognized phenotypes driven by vascular dysfunction [23,30].

### 5.2. Computational Integration of Multidomain Data

Future progress in precision medicine is likely to depend on computationally enabled integration of multidomain datasets rather than isolated use of conventional clinical descriptors [23,31,34,36]. Clinical variables, biomarkers, advanced imaging, vascular markers, and emerging omics data can be combined to identify mechanistically coherent patient clusters and support a transition from descriptive classification toward mechanism-based disease taxonomy [31,36].

**Figure 5:**
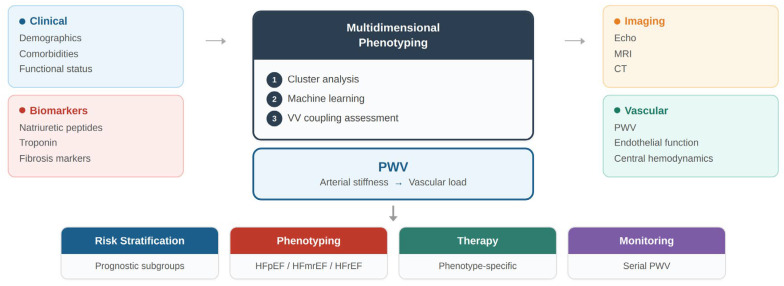
Multidimensional phenotyping and clinical integration of PWV in heart failure.

A conceptual framework illustrating the integration of vascular functional markers—particularly pulse wave velocity—into multidimensional precision phenotyping of heart failure is presented in Figure 5 [23,44].

Integrated phenotyping combines clinical data, biomarkers, imaging, and vascular assessment with computational approaches to identify distinct heart failure phenotypes. PWV provides complementary information on arterial stiffness and vascular load, supporting risk stratification, phenotype identification, targeted therapy, and longitudinal monitoring. Such integrative models may help bridge the gap between vascular physiology and precision cardiovascular medicine.

Key data streams relevant to integrated cardiovascular phenotyping include the following:

- Clinical characteristics and comorbidities;
- Circulating biomarkers reflecting myocardial stress, inflammation, and fibrosis;
- Advanced cardiac imaging parameters;
- Genomic and proteomic information;
- Vascular function markers.

Combining these dimensions through advanced computational models may enable the identification of clinically meaningful patient clusters characterized by shared pathophysiological mechanisms [31,36].

Machine learning approaches have already demonstrated the potential to uncover previously unrecognized HF phenotypes with distinct clinical trajectories [8]. For example, unsupervised machine learning approaches such as phenomapping have been used to identify distinct HFpEF subgroups based on clinical, imaging, and biomarker data, enabling improved risk stratification and targeted therapeutic strategies [8,35,69]. However, most existing models still rely heavily on myocardial parameters and systemic biomarkers. Vascular parameters are rarely included despite their fundamental physiological role [10].

Incorporating markers of arterial stiffness—particularly PWV—into such models may significantly enhance phenotypic resolution [17,19].

Concrete steps toward clinical validation could include prospective multicenter cohort studies in which PWV and complementary vascular markers are collected alongside standard clinical, imaging, and biomarker data in broadly phenotyped heart failure populations. Unsupervised clustering algorithms could then be applied to identify vascular-dominant phenotypes, followed by external validation in independent registries. Furthermore, the development of interpretable machine learning models—such as gradient-boosted decision trees incorporating PWV alongside established predictors—could facilitate clinical adoption by providing transparent risk scores amenable to integration into electronic health record-based decision support tools.

Such integrated phenotyping frameworks could ultimately facilitate the transition from descriptive HF classifications toward mechanism-based disease taxonomy [23,31].

### 5.3. The Concept of a Vascular Phenotype of Heart Failure

One intriguing hypothesis emerging from recent research is the existence of a vascular-driven phenotype of HF, particularly within the HFpEF spectrum [9,12].

Patients with HFpEF frequently exhibit a constellation of features suggestive of accelerated vascular aging, including the following:

- Increased arterial stiffness;
- Systemic hypertension;
- Endothelial dysfunction;
- Microvascular rarefaction;
- Metabolic and inflammatory abnormalities [9,25].

These alterations contribute to increased pulsatile hemodynamic load and impaired ventricular relaxation [48]. In this context, the myocardium may be viewed not as the primary origin of disease but rather as the organ most vulnerable to chronic vascular stress.

This perspective raises the possibility that certain HF phenotypes may be driven primarily by vascular pathology rather than intrinsic myocardial dysfunction. If confirmed, such insights would have important therapeutic implications [12,22,39,70,71].

For example, therapies targeting vascular stiffness, endothelial function, or microvascular perfusion could potentially play a more prominent role in selected HF subgroups [23,30].

### 5.4. Pulse Wave Velocity as a Tool for Risk Stratification and Monitoring

Beyond its role as a marker of arterial stiffness, PWV may also serve as a clinically useful tool for risk stratification and disease monitoring in HF.

Several characteristics make PWV particularly attractive in this context.

(a) Physiological relevance

PWV reflects the pulsatile component of arterial load, which directly influences ventricular work and myocardial oxygen demand [17,47].

(b) Prognostic value

Numerous studies have demonstrated that increased PWV independently predicts cardiovascular events and mortality [19,59,64].

(c) Non-invasive assessment
(d) PWV can be measured rapidly and non-invasively using widely available technologies [57,58,63]. In clinical practice, carotid–femoral PWV is typically obtained within less than five minutes using validated automated devices such as SphygmoCor (AtCor Medical) or Complior (Alam Medical), which record simultaneous or sequential pressure waveforms at two arterial sites, requiring minimal operator training and no patient preparation beyond a brief rest period. The algorithm of the SphygmoCor takes into account vascular remodeling.

Changes in PWV may reflect modifications in vascular structure and function over time [17,54].

These features suggest that PWV could potentially be incorporated into clinical algorithms aimed at identifying HF patients at increased risk of disease progression.

Moreover, serial assessment of PWV might provide insights into the vascular effects of therapeutic interventions. Several pharmacological agents used in HF—including renin–angiotensin system inhibitors, mineralocorticoid receptor antagonists, and sodium–glucose cotransporter-2 inhibitors—have been shown to influence vascular function and arterial stiffness [47,55,72].

Monitoring PWV could therefore help evaluate treatment response beyond conventional clinical endpoints.

From a practical standpoint, integration of PWV assessment into routine heart failure evaluation could follow a stepwise approach. In outpatient clinics, automated oscillometric devices allow PWV measurement to be performed alongside standard vital signs, requiring no specialized training. A pragmatic implementation model could include PWV assessment at initial HF diagnosis, at each major therapeutic adjustment, and at regular intervals during follow-up. Abnormal PWV values could trigger referral for comprehensive vascular evaluation and guide the selection of therapies known to improve arterial compliance, such as renin–angiotensin system inhibitors or SGLT2 inhibitors. Clinical decision support systems could incorporate PWV thresholds alongside established parameters to generate individualized risk profiles and treatment recommendations [51,54,61,73]. In addition, PWV assessment may be particularly informative in specific heart failure phenotypes. Patients with HFpEF and multiple cardiometabolic comorbidities, including hypertension, obesity, and diabetes, are more likely to exhibit a vascular-driven phenotype characterized by increased arterial stiffness and impaired ventricular–vascular coupling. In such cases, elevated PWV values may support a more aggressive approach to risk factor modification and therapeutic optimization, including stricter blood pressure control and the use of therapies with known vascular effects. Moreover, PWV may provide incremental prognostic information and contribute to more refined risk stratification beyond conventional clinical and imaging parameters [47,56,57,59,60].

### 5.5. Future Research Directions

Despite the growing interest in vascular contributions to HF pathophysiology, several important questions remain unanswered.

Moreover, it should be acknowledged that existing evidence linking arterial stiffness to heart failure outcomes is not entirely consistent. Some studies have reported attenuated associations after adjustment for conventional cardiovascular risk factors, and the independent prognostic value of PWV beyond established markers such as natriuretic peptides remains debated. Methodological differences in PWV assessment—including variations in device type, measurement site, and path length estimation—further complicate cross-study comparisons and limit the generalizability of current findings. Additionally, the vascular phenotype concept in HFpEF, while compelling, has not yet been validated in prospective interventional studies targeting arterial stiffness as a modifiable therapeutic endpoint.

First, the precise role of arterial stiffness in the initiation and progression of HF remains incompletely understood. Longitudinal studies are needed to determine whether increased PWV represents merely a marker of cardiovascular risk or a causal contributor to disease development [19,54].

Second, the integration of vascular parameters into computational phenotyping models requires further investigation. Large-scale datasets combining clinical, imaging, biomarker, and vascular measurements will be necessary to fully explore the potential of integrated cardiovascular phenotyping [23,31,34,35].

Third, interventional studies are needed to evaluate whether therapeutic strategies targeting vascular stiffness can improve outcomes in specific HF phenotypes.

Advances in digital health technologies and wearable sensors may further facilitate continuous monitoring of vascular function and hemodynamic parameters, opening new avenues for personalized cardiovascular care [74].

### 5.6. Limitations of Current Evidence

Despite the growing body of evidence linking arterial stiffness with heart failure pathophysiology, several limitations should be acknowledged. First, most available data are derived from observational studies, which limits the ability to establish causal relationships between arterial stiffness and heart failure development or progression. Second, methodological heterogeneity in pulse wave velocity measurement techniques—including differences in devices, acquisition protocols, and path length estimation—may complicate comparisons across studies. Third, vascular parameters are still rarely incorporated into large-scale phenotyping studies and machine learning models, which limits the integration of arterial stiffness into current precision medicine frameworks. Future prospective studies integrating vascular, myocardial, and systemic parameters will be necessary to clarify the mechanistic role of arterial stiffness and its potential therapeutic implications in heart failure.

## 6. Conclusions

Heart failure represents a complex and heterogeneous syndrome that extends beyond myocardial dysfunction, encompassing dynamic interactions between cardiac, vascular, and systemic factors. The integration of vascular function into precision medicine frameworks offers an opportunity to refine phenotypic classification and improve mechanistic understanding of the disease.

Arterial stiffness, as assessed by pulse wave velocity, represents a robust and physiologically relevant marker of vascular aging and cardiovascular risk. Its incorporation into multimodal phenotyping strategies may enhance risk stratification and support the identification of clinically meaningful subgroups within the heterogeneous spectrum of heart failure.

Future research integrating myocardial, vascular, and systemic domains through advanced computational approaches is likely to redefine heart failure classification and pave the way toward truly personalized cardiovascular medicine.

## Figures and Tables

**Table 1:** Core domains in precision medicine-based phenotyping of heart failure.

Domain	Representative Variables	Pathophysiological Contribution	Clinical Utility	Methodological Approach
Clinical profiling	Age, sex, HF etiology, comorbidities (hypertension, diabetes, and obesity), and NYHA class	Reflects patient-level heterogeneity and cumulative disease burden	Baseline characterization, risk stratification, and identification of clinical phenotypes	Clinical assessment and registry-based data
Circulating biomarkers	BNP, NT-proBNP, cardiac troponins, galectin-3, and soluble ST2	Reflect myocardial stress, injury, fibrosis, and inflammation	Diagnostic support, prognostic stratification, and therapy monitoring	Laboratory assays and biomarker panels
Cardiac imaging	Echocardiography (LVEF, diastolic parameters, and global longitudinal strain), cardiac MRI, and cardiac computed tomography (functional CT, late iodine enhancement, and extracellular volume quantification)	Characterizes myocardial structure, function, and tissue remodeling	Phenotype definition, detection of subclinical dysfunction, and guiding therapy	Imaging-based quantitative assessment (echocardiography, CMR, and cardiac CT)
Computational phenotyping	Cluster analysis, machine learning, and phenomapping approaches	Identifies latent patient clusters based on multidimensional data integration	Discovery of novel HF phenotypes and prognostic subgroups	Unsupervised learning and data-driven modeling
Vascular functional markers	Pulse wave velocity, arterial stiffness indices, and wave reflection parameters	Reflect arterial load, vascular aging, and ventricular–vascular interaction	Complements myocardial assessment and enhances phenotypic resolution	Non-invasive vascular assessment (cfPWV and oscillometric methods)

Legend: HF, heart failure.

**Table 2:** Representative clinical studies evaluating pulse wave velocity and arterial stiffness in heart failure.

Study	Population	Study Design	PWV Assessment	Main Focus	Principal Findings	Clinical Relevance
Weber et al. [54]	Patients with chronic heart failure	Observational cohort study	cfPWV/arterial stiffness indices	Prognostic significance of arterial stiffness	Increased arterial stiffness, associated with impaired hemodynamics and worse clinical status	Supports role of PWV as marker of disease severity
Chirinos et al. [47]	Patients with HFpEF and abnormal ventricular–vascular coupling	Mechanistic observational study	cfPWV and wave reflection indices	Ventricular–vascular interaction	Increased arterial stiffness, associated with impaired coupling and adverse hemodynamics	Highlights vascular contribution in HFpEF
Pandey et al. [60]	Community-based population	Prospective cohort study	PWV	Association with incident HF	Higher PWV, associated with increased risk of incident HF	Identifies PWV as early risk marker
Shah et al. [8]	HFpEF phenotyping cohorts	Machine learning/phenomapping study	Multimodal [including vascular parameters]	Data-driven HF phenotyping	Identification of distinct HF phenotypes with different clinical trajectories	Supports integration of vascular markers in phenotyping
Vlachopoulos et al. [19]	Cardiovascular populations	Systematic review and meta-analysis	PWV	Prognostic value of arterial stiffness	Each 1 m/s increase in PWV, associated with increased CV events and mortality	Strong evidence for prognostic role of PWV
Laurent et al. [17]	Vascular research consensus	Expert consensus document	cfPWV	Methodological standardization	cfPWV established as gold standard for arterial stiffness	Provides methodological framework

Abbreviations: PWV, pulse wave velocity; HF, heart failure.

## Data Availability

The original contributions presented in this study are included in the article. Further inquiries can be directed to the corresponding author(s).

## Abbreviations

The following abbreviations are used in this manuscript:

HF	heart failure
HFrEF	heart failure with reduced ejection fraction
HFpEF	heart failure with preserved ejection fraction
PWV	pulse wave velocity
cfPWV	carotid–femoral pulse wave velocity
CMR	cardiac magnetic resonance
